# A cross-border comparison reveals expanded microbial diversity and zoonotic risk in *Rhipicephalus microplus* ticks of Yunnan, China

**DOI:** 10.1186/s13071-026-07572-1

**Published:** 2026-08-13

**Authors:** Yayu Fan, Guowang Liu, Genggeng Liang, Xianyao Yang, Jianyang Zhang, Ping Sun, Li Liu, Lingling Ye, Xueshan Xia, Yue Feng

**Affiliations:** 1 Faculty of Life Science and Technology and Yunnan Provincial Key Laboratory of Public Health and Biosafety, Kunming University of Science and Technology, Kunming, China; 2 Yunnan Kecan Biotechnology Co., Ltd., Kunming, China; 3 Kunming Customs Technical Center, Kunming Customs District P.R. China, Kunming, China; 4 Yunnan Provincial Key Laboratory of Public Health and Biosafety, Kunming Medical University, Kunming, Yunnan China

**Keywords:** Microbial diversity, Virome, Bacteriome, Zoonotic potential, Meta-transcriptomes, 16S rRNA

## Abstract

**Background:**

*Rhipicephalus microplus* is a critical vector for numerous agents of livestock and human diseases. Border regions are potential hotspots for pathogen exchange and emerging tick-borne diseases, yet comprehensive surveys of the total pathogen community (virome and microbiome) in ticks from these areas are limited. This study aimed to characterize the microbial diversity within *R. microplus* across different geographical locations (local vs. imported) and tissue types in Yunnan, China, to understand ecological drivers and assess associated zoonotic risks.

**Methods:**

We performed meta-transcriptomic sequencing to profile the RNA virome and 16S rRNA gene sequencing to analyze the bacterial microbiome of *R. microplus* samples. Ticks were dissected into three key tissues: midgut, salivary glands, and ovaries. Bioinformatic and statistical analyses included taxonomic assignment, diversity metrics (alpha and beta diversity), co-occurrence network analysis, and tests for assessing the impact of geographic origin and tissue type on community structure.

**Results:**

Our analysis revealed a highly diverse community of virome and bacterial microbiome. The virome comprised 63 viral families, including 28 with known animal hosts, such as the clinically significant families Flaviviridae, Phenuiviridae, and Peribunyaviridae. The bacterial microbiome included 167 bacterial genera, dominated by known pathogenic genera (*Coxiella*, *Rickettsia*, *Anaplasma*, *Ehrlichia*). We identified three novel viruses. Viral diversity was significantly richer in cross-border than in local samples, and the midgut exhibited the highest viral diversity among tissues. Network analysis indicated potential interactions between specific viral (e.g., *Chuviridae*) and bacterial taxa.

**Conclusions:**

This study reveals that *R. microplus* in border areas harbors a high diversity of pathogens with zoonotic potential. The composition of this pathogen community is fundamentally shaped by both geographic origin and vector tissue tropism. The elevated diversity at cross-border sites identifies them as key surveillance targets. These findings provide crucial data for informing surveillance strategies and early warning systems against tick-borne diseases in high-risk border regions.

**Graphical Abstract:**

**Figure:**
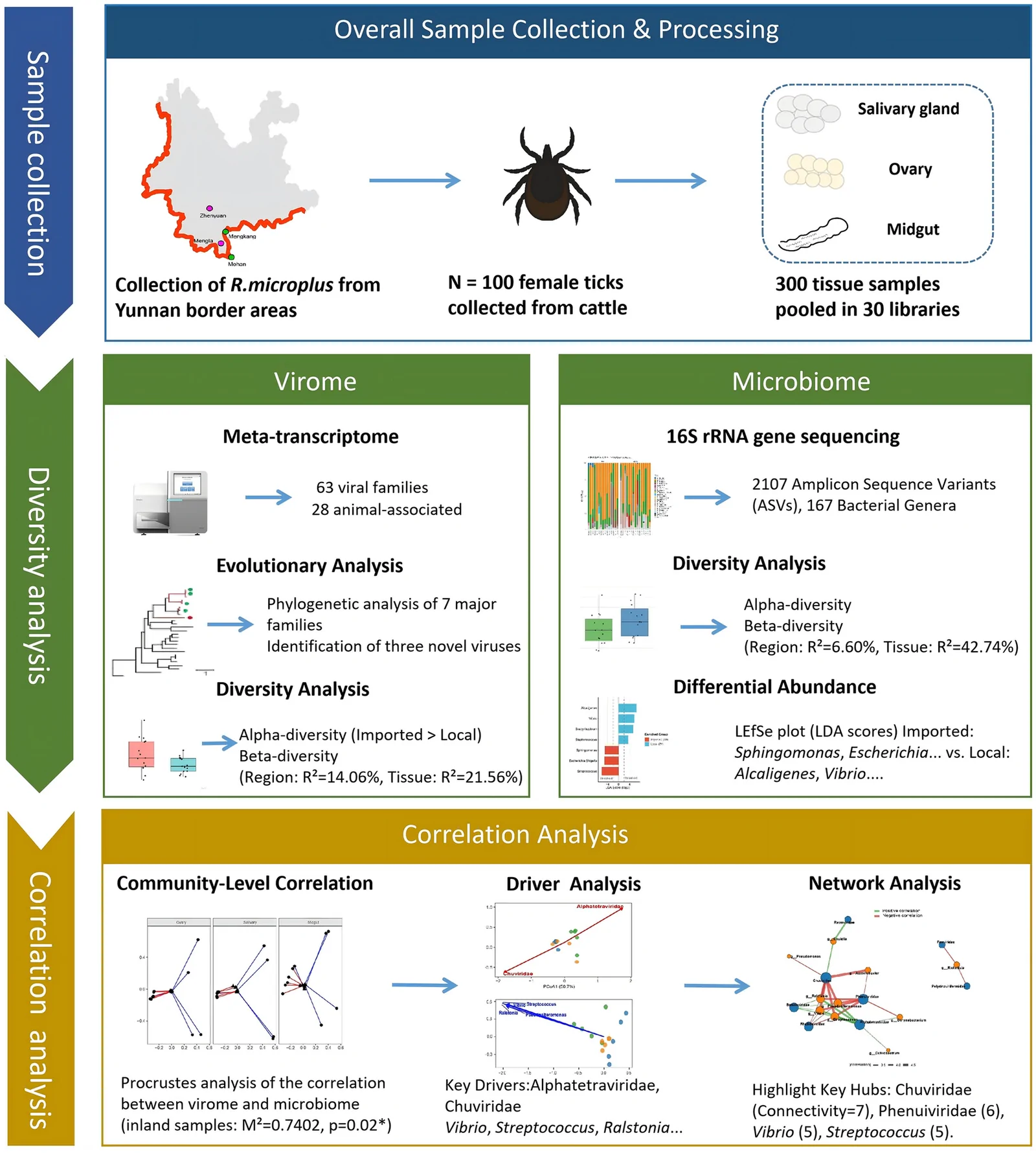

**Supplementary Information:**

The online version contains supplementary material available at https://doi.org/10.1186/s13071-026-07572-1.

## Background

Ticks (Acari: *Ixodida*) are blood-feeding ectoparasites. They play a crucial role as vectors for a wide range of pathogens, including viruses, bacteria, and protozoa. This poses a significant threat to global public and veterinary health [1]. For example, the severe fever with thrombocytopenia syndrome virus (SFTSV), carried by the tick species *Haemaphysalis longicornis*, causes fatal hemorrhagic fever in humans with mortality rates exceeding 30% in China [2]. Similarly, the bacterium *Borrelia burgdorferi*, which causes Lyme disease, is carried by the tick *Ixodes scapularis* and affects humans and livestock throughout Eurasia and North America [3]. Currently, 105 tick-borne viruses are associated with human and animal infections or exposure. Of these, 59 are confirmed to cause diseases such as Crimean-Congo hemorrhagic fever, fever with thrombocytopenia syndrome, and forest encephalitis [4]. However, aside from a few virus species, such as the Flaviviridae*,* Nairoviridae*,* and Phenuiviridae families, the public health significance and zoonotic risks of many other viruses remain unclear. Furthermore, research indicates that climate change and environmental alterations are causing tick species to expand their geographical range [5, 6]. This expansion contributes to the spread of tick-borne pathogens and an increase in related disease incidence [6, 7].

The discovery of tick-borne pathogens has increased significantly due to the use of next-generation sequencing (NGS) technologies, which are highly sensitive and fast [8, 9]. For instance, researchers sequenced the meta-transcriptomes of 31 tick species belonging to the Argasidae and Ixodidae families in mainland China, and they identified 724 RNA viruses and revealed distinct virome compositions among different genera [10]. These findings greatly enriched the baseline data on tick-borne viruses in China. Additionally, a meta-transcriptomic analysis of ticks from Yunnan Province revealed 13 novel RNA viruses, including members of the Phenuiviridae and Nairoviridae families. The potential pathogenicity of these viruses is unknown [11]. Similarly, Jingmen tick virus (JMTV), a segmented flavivirus, was identified in *Haemaphysalis longicornis* ticks and was later linked to human febrile illnesses via NGS [12, 13].

Yunnan’s border regions, which border Myanmar, Laos, and Vietnam, are biodiversity hotspots and critical zones for the cross-border transmission of pathogens [14]. Frequent transboundary activities, such as livestock trade, human migration, and wildlife movement, facilitate pathogen spillover. For example, distinct genotypes of SFTSV were found in ticks that infested cattle imported from Myanmar, indicating the cross-border introduction of the virus [15]. Migratory ungulates, such as black muntjac deer (*Muntiacus crinifrons*), carry *Borrelia* and *Anaplasma* species, which spread pathogens across ecosystems [16, 17]. The widespread use of high-throughput sequencing technologies to discover tick-borne pathogens has led to the identification of a growing number of novel pathogens. However, analyses of the tick microbiome in specific regions, particularly border areas, remain limited despite the increased risk of cross-border transmission of these pathogens. Most studies focus exclusively on bacteria or viruses and lack comparative and integrative approaches [10, 18–20]. Furthermore, although different tick tissues (e.g., the midgut, salivary glands, and ovaries) play distinct roles in pathogen transmission, research on tissue-specific microbial distributions is still in its early stages [21, 22]. Overall, current research on tick-associated microbiomes shows significant gaps in comprehensiveness, spatial dimension, and organizational depth.

## Methods

### Sample collection

**Fig. 1:**
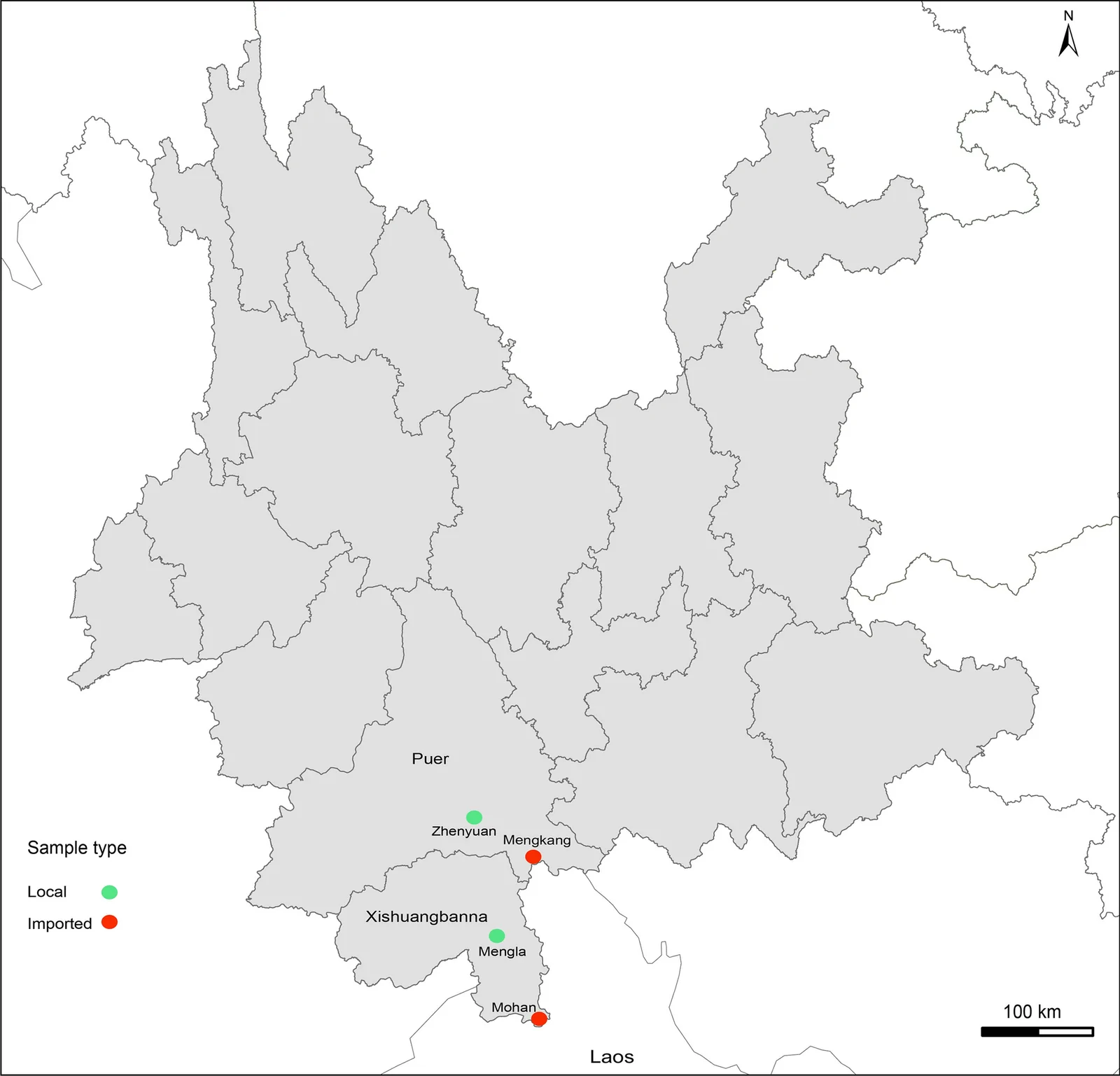
Geographic map of sample collections in the Yunnan region, China. The color of the circle represents the type of tick samples collected. Green for samples collected from local cattle in Mengla Country and Zhenyuan County, red for samples collected from imported cattle in Mohan and Mengkang Port

Adults of tick samples were captured directly from the body surface of cattle using curved tweezers in the Mohan Port (101° 68' E, 21° 18' N), Mengkang Port (101° 71' E, 22° 49' N), Mengla Country (101° 29' E, 22° 17' N), and Zhenyuan County (101° 20' E, 23° 65' N) in 2024 (Fig. 1). Of these, samples collected from imported cattle in Mohan and Mengkang Port are classified as imported, and samples collected from local cattle in Mengla Country and Zhenyuan County are classified as local. Species identification was conducted using morphological characteristics of capitulum, palps, scutum, coxae, and tarsi under a stereomicroscope by a professional technician [23]. And a fragment of the cytochrome C oxidase subunit I (COI) gene was amplified by polymerase chain reaction (PCR) for molecular identification (primers: COI-F: 5′-GCTCAACAAAYCATAAAGACATTGG-3′ and COI-R: 5′-CTTCAGGRTGGCCAAARAATC-3′) [20]. Thermal cycling conditions were: a predenaturation step at 95 °C for 3 min, followed by 40 cycles of denaturation for 30 s at 95 °C, annealing for 30 s at 50 °C, and extension for 60 s at 72 °C, ending with 5 min at 72 °C. After identification, the ticks were immersed in RNAlater buffer and stored at − 80 °C.

### RNA extraction and library preparation for meta-transcriptomic sequencing

Among the total ticks collected, 100 live adult female ticks were selected from 19 cattle, and dissection of ticks was conducted using a stereomicroscope [24]. Ticks from the imported or local sampling locations were assigned to five groups, respectively. Each tick was washed three times using 75% ethanol, and followed by three washes with sterile phosphate-buffered saline (PBS; pH 7.2) [21]. All ticks within a single group were dissected simultaneously under a microscope to collect the midgut, salivary glands, and ovaries. Tissues from the same group were pooled by tissue type, yielding one pooled sample per tissue and three pools per tick group, resulting in a total of 30 distinct sample groups, as summarized in Table S1.

Each group of samples was homogenized using an RLT solution in the frozen mortar treated with liquid nitrogen and then centrifuged at 5,000 × g for 20 min at 4 °C. The supernatant was collected and stored at − 80 °C until further processing. Total RNA from each pool was extracted and purified using the RNAsimple Total RNA Kit (TIANGEN, China) following the manufacturer’s instructions for meta-transcriptomic sequencing. A negative control was included in parallel with the tick samples. Meta-transcriptome library construction and ribosomal RNA depletion were carried out using the VAHTS® Universal V8 RNA-seq Library Prep Kit for Illumina (Vazyme, China), and paired-end (2 × 150 bp) sequencing of each RNA library was performed using an Illumina NovaSeq 6000 System (MEGAN, China).

### DNA extraction and library preparation for 16S rRNA gene sequencing

Separate aliquots were used for RNA and DNA extraction, as summarized in Table S1. Total DNA for each pool was also extracted and purified using the Tiangen Blood/Cell/Tissue Genomic DNA Extraction Kit following the manufacturer’s instructions for 16S rRNA gene sequencing. Bacterial 16S rRNA gene amplification was carried out using 338F/806R universal primers in the V3–V4 region (338-F: 5’-ACTCCTACGGGAGGCAGCA-3’ and 806-R: 5’-GGACTACHVGGGTWTCTAAT-3’) [25]. Negative and positive controls were processed in parallel to monitor contamination and reaction consistency. Libraries were prepared using the NEBNext UltraTM DNA Library Prep Kit for Illumina (NEB, USA) following the manufacturer’s recommendations. Paired-end (PE) sequencing of each library was subsequently performed on the Illumina NovaSeq 6000 (MEGAN, China).

### Viral contig assembly and annotation

Raw paired-end sequence reads obtained through meta-transcriptomic sequencing were first quality controlled using Trimmomatic (v039) [26] to remove adapter sequences, low-quality bases, and duplicate reads. Subsequently, the cleaned reads were compared to the SILVA database (Silva.132) to eliminate reads related to rRNA using Bowtie2 (v2.5.4) [27]. Then, the non-rRNA reads were compared with the *R. microplus* genome (GCF_043290135.1) using Bowtie2 (v2.5.4) [27] to remove reads associated with the tick genome. The unmapped reads were subsequently assembled de novo into contigs using MEGAHIT (v.1.2.9) with default parameter settings [28]. All the assembled contigs with a length of above 300 bp were then aligned against the NCBI Non-Redundant Protein Sequence Database (NR, downloaded on October 27, 2024) using Diamond blastx (v2.1.8) [29], with an e-value threshold of 1 × 10^−5^ to maintain high sensitivity at a low false positive rate. Contigs with the top BLAST hit classified under the kingdom ‘viruses’ were preliminarily identified as viral sequences, and were further compared against the NCBI nucleotide sequence database using BLASTN, with an e-value threshold of 1 × 10^−5^ [30]. Viral sequence classification was conducted according to the International Committee on Taxonomy of Viruses (ICTV) reports (https://talk.ictvonline.org/ictv-reports/ictv_online_report/). Contigs whose best match lacked assignment to a defined virus family were designated as ‘unclassified’. Subsequently, based on the annotated family-level classification, putative viral hosts were inferred primarily using the ViralZone resource (www.expasy.org/viralzone/). Contigs associated with known vertebrate and/or invertebrate virus groups were retained, whereas those clustering with bacterial, fungal, or plant virus groups were excluded. Finally, the putative vertebrate and/or invertebrate host associations for the retained viral contigs were further corroborated through phylogenetic analysis with viruses of known host origin. The NCBI ORFfinder was used to search each of the viral genome open reading frames (https://ftp.ncbi.nlm.nih.gov/genomes/TOOLS/ORFfinder/). A comparative analysis of the nucleotide sequences of the genomes and the amino acid sequences of ORFs was conducted by comparing the sequences with those of related viruses available in GenBank.

### Quantification of viral abundance

To quantify viral abundance, the non-rRNA reads from each library were mapped to the viral contigs using Bowtie2 with sensitive parameters [27]. SAMtools (v1.21) was employed to sort and index the alignments [31], enabling the extraction of read counts per contig. These counts were normalized as reads per million non-rRNA reads (RPM). A threshold of RPM ≥ 1 was applied to filter out potential false-positive contigs [32]. This processed data was then used to estimate the relative abundance of each viral family per library and a heatmap for visualizing the virome profile was plotted using the R software (pheatmap package, v1.0.13) [32].

### Bioinformatic analysis of the 16S rRNA gene sequencing data

The raw FASTQ files were subsequently subjected to demultiplex using the Quantitative Insights into Microbial Ecology (QIIME2) pipeline [33]. Amplicon sequence variants (ASVs) were obtained after quality control, paired-end reads merging, and chimeric sequences deletion conducted using the DADA2 plugin of QIIME2 [34]. A Naive Bayes classifier was trained using Silva database 138–99 (https://data.qiime2.org), and taxonomic classification of representative sequences was performed employing the feature-classifier plugin of QIIME 2 [35]. Taxonomic information on bacteria from phylum to species was obtained in all samples.

### Phylogenetic analysis

The sequences identified in this study were aligned with related reference sequences downloaded from GenBank with the ClustalW program in MEGA X [36], based on the sequence variants from this study and other closely related sequences obtained from the NCBI database by BLASTn analysis. The optimal model for nucleotide substitution was identified with jModeltest (v.2.1.10) [37]. Maximum likelihood (ML) trees were inferred using the MEGA-X program with the model (GTR + G + I) and 1,000 bootstrap replicates for each family of viruses and each genus of bacteria we focused on.

### Analysis of variations in virus and bacteria diversity between locations and tissues

Viral and bacterial communities were analyzed separately. For alpha diversity analysis of bacteria, the Shannon diversity index was calculated according to the generated genus-level abundance table using the R software (vegan package, v2.5-7) [38], which takes into account of both richness and evenness of microbial communities. Statistical comparison of alpha diversity (the Shannon diversity index) of microbiome between different levels of variables (locations and tissues) was performed using t-test or Wilcoxon rank-sum test, Kruskal–Wallis test, and the Bonferroni correction was applied to adjust the significance levels accordingly.

Beta diversity analysis was calculated with Bray–Curtis distance matrices to evaluate differences between the microbial communities of our samples using the R software (vegan package, v2.5-7) [38], and the results were plotted using nonmetric multidimensional scaling (NMDS), using the R software (ggplot2 package, v3.5.2) [18]. Additionally, PERMANOVA analysis was applied to detect differences in microbial community structure among different groups of locations and tissues, using the R software (vegan package, v2.5-7) [38].

Linear Discriminant Analysis Effect Size (LEfSe) was performed using LEfSe software [39] to reveal features (bacteria) that most likely explained differences among different groups of locations and tissues, with an LDA score filter value of 2. Statistical comparisons between groups were performed using the Wilcoxon rank-sum test, with a significance threshold set at *P* < 0.05.

### Analysis of the correlation between virus diversity and bacteria diversity

Procrustes analysis was implemented to ascertain the correlation between virus and bacteria diversity, as measured by the Bray–Curtis distance matrix, employing the R software (vegan package, v2.5-7) [38]. The resultant data were subsequently displayed using the R software (ggplot2 package, v3.5.2) [18]. The envfit analysis was utilized to identify viral families that exert a substantial influence on microbial community structure, as well as bacterial genera that demonstrate a significant impact on viral community structure using the R software (vegan package, v2.5-7) [38]. Furthermore, Spearman correlation analysis was conducted to ascertain significant virus–microbe correlations, and Key correlation networks were subsequently constructed using the R software (psych package, v2.5.6) [40].

## Results

### Overview of the virome and bacterial microbiome

#### Tick collection and identification

Among the total of ticks collected, 100 live female ticks were selected, which were identified as *R. microplus* through species identification. Sequence similarity threshold of ≥ 99% was used to define species-level classification [41]. All representative COI sequences have been deposited in NCBI GenBank under accession numbers PZ457797–PZ457827. And a total of 300 tissue samples, encompassing salivary glands, midguts, and ovaries, were obtained through a dissection conducted using a stereomicroscope. 30 libraries were pooled for meta-transcriptomic sequencing and 16S rRNA gene sequencing.

### Annotation of viruses and bacteria

**Fig. 2:**
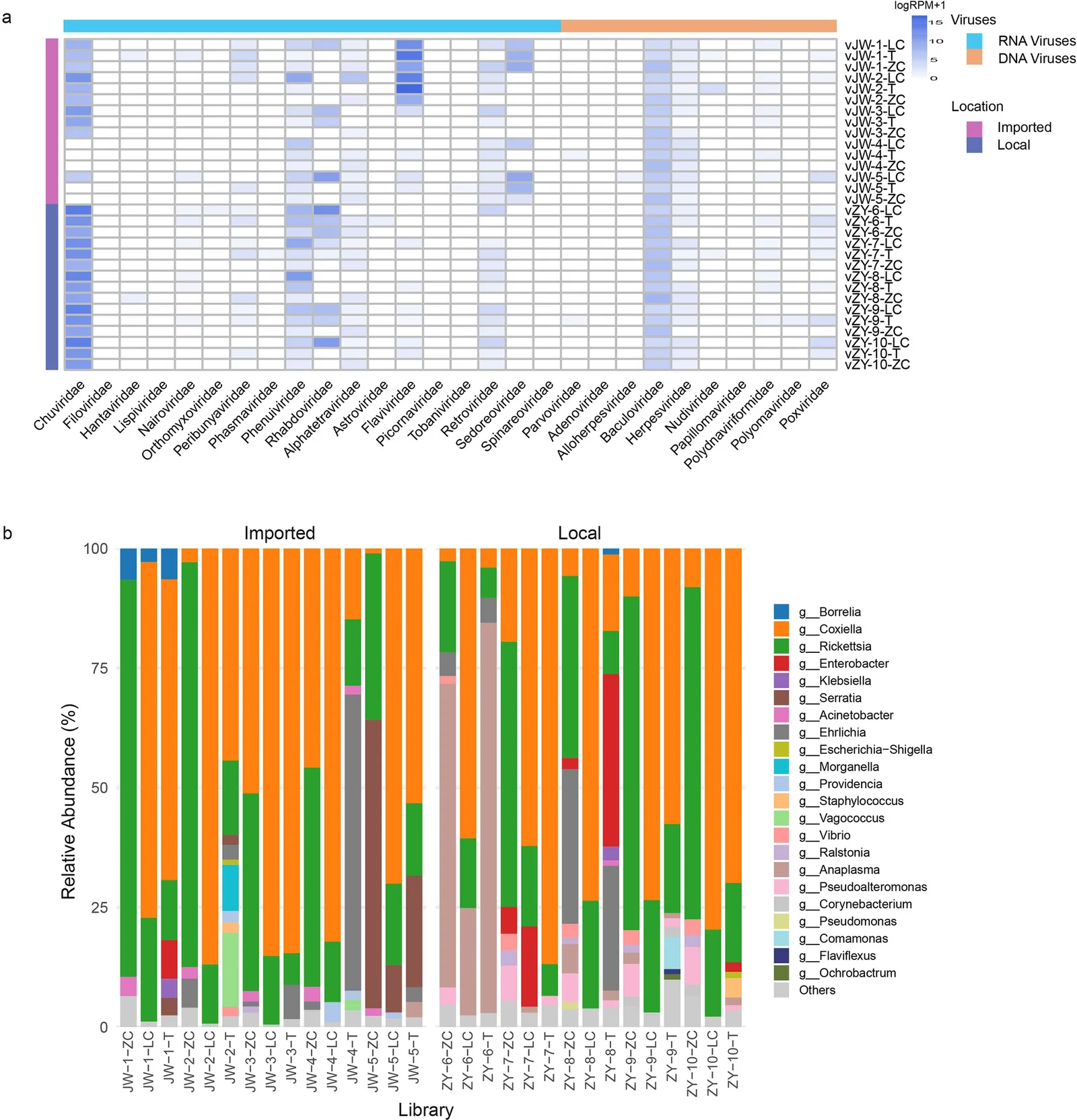
Overview of the virome and microbiome annotated in this study. The name of library: “-LC”, ovaries; “-T”, salivary glands; “-ZC”, midguts. **a** Heatmap based on the normalized numbers of sequence reads for 28 families of animal viruses in each pooled sample. The relative abundance of each cluster was calculated as log2 (RPM + 1). **b** Bar graph depicting the bacterial amplicon sequence variants (ASVs) composition at the genus level for each library. “Local” for samples collected from local cattle in Mengla Country and Zhenyuan County, “Imported” for samples collected from imported cattle in Mohan and Mengkang Port

A total of 498.34 gigabytes (GB) of raw data from meta-transcript sequencing (150 base pairs in length), including 3.32 × 10^9^ raw reads, were obtained. Subsequent to the implementation of quality control measures and the elimination of rRNA and *R. microplus* genome reads, a total of 5.97 × 10^6^ viral reads were identified, constituting 2.21% of the total clean reads. The percentage of viral reads in each library ranged from 0.02% to 16.04% (Supplemental Table S1). A total of 918 viral contigs were assembled by de novo assembly, with lengths ranging from 300 to 17,528 base pairs (bp). A total of 63 viral families were annotated, including 28 animal-associated viral families, including several important zoonotic viruses, such as the families Flaviviridae, Nairoviridae, and Phenuiviridae. Among these, the families Baculoviridae, Chrysomelidae, and Herpesviridae exhibited a high degree of distribution. The families Flaviviridae and Sedoreoviridae are predominantly present in imported samples (Fig. 2a).

Additionally, 1.92 gigabytes (GB) of raw data, including 6.56 × 10^6^ raw reads, were obtained from 16S rRNA V3–V4 region sequencing. Following the processes of clustering, the elimination of chimeric sequences, and filtration, a total of 2,809,054 sequences were deemed suitable for analysis (Supplemental Table S2). The median sequencing output was 94,928 reads. The saturation plateau of the rarefaction curve was used to determine a sampling depth of 55,000, which was selected to ensure the retention of the maximum number of samples while also capturing an adequate bacterial diversity for comparative analysis between samples. In the aftermath of rarefaction, a total of 2,107 ASVs in 30 samples were subjected to taxonomic classification, with sequences designated as mitochondria or chloroplasts being excluded from the analysis. This process resulted in the identification of 167 genera (level 6 classification) from the samples that were included in the diversity analysis. Among them, seven genera accounted for the highest proportion (> 1% of the total count), including *Coxiella* (45.95%), *Rickettsia* (28.19%), *Anaplasma* (7.74%), *Ehrlichia* (4.55%), *Serratia* (2.94%), *Enterobacter* (2.41%), and *Pseudoalteromonas* (1.51%) (Fig. 2b).

## Viral diversity, association with tissue and location

### Alpha diversity

**Fig. 3:**
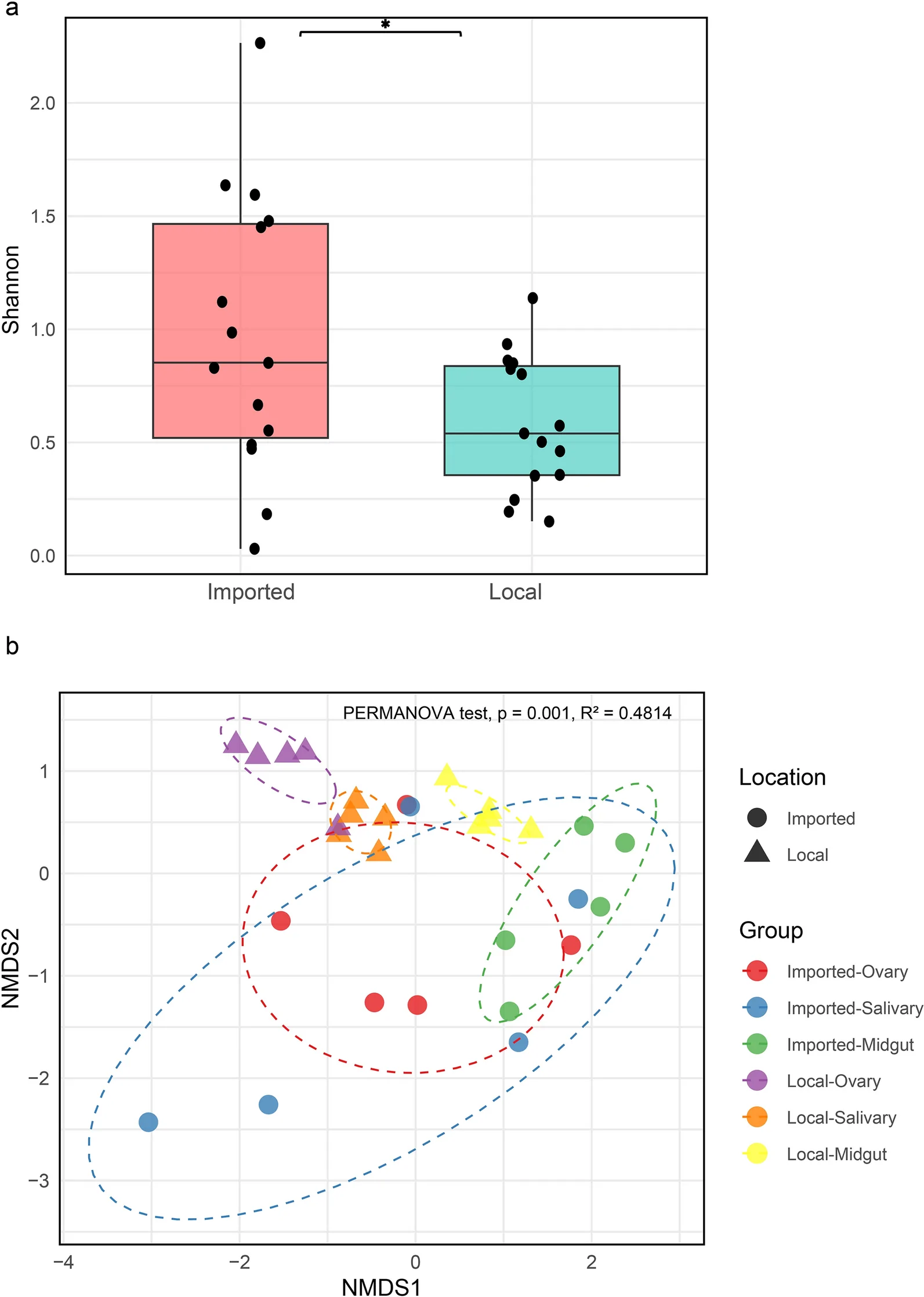
Regional and tissue variation of the virome. **a** Geographical difference in total virus diversity (Shannon diversity index). *P < 0.05. **b** Non-metric multidimensional scaling analysis displays the variations in virus composition between regions and tissues. In the NMDS plots, the circles represent the 95% normal probability ellipse. *T*-tests were performed for each pairwise comparison

The mean Shannon index for imported samples was significantly higher than that for local samples (*T*-test, *p* < 0.05), indicating higher viral diversity in imported samples (Fig. 3a). At the tissue level, Shannon diversity of midgut tissue exhibited the highest levels of diversity, while salivary α diversity demonstrated the lowest levels of diversity. However, no significant differences in Shannon diversity were observed among different tissues (ovary, salivary, midgut) (Additional file 1: Fig. S1a), nor among the six regions-tissue groups (Additional file 1: Fig. S1b).

### Beta diversity

A substantial discrepancy in the virome was identified among distinct geographical regions and anatomical tissues (PERMANOVA test, *p* = 0.001, *R*^2^ = 0.4814). This finding indicates that the composition of the total virus differs significantly between samples from imported and local regions (PERMANOVA test, *p* = 0.001, *R*^2^ = 0.1406) and among ovary, salivary gland, and midgut tissues (PERMANOVA test, *p* = 0.001, *R*^2^ = 0.2156). In summary, both region and tissue have been demonstrated to significantly influence virus community structure (*β* diversity), with the factor of tissue explaining a higher proportion (*R*^2^ = 21.56% vs. *R*^2^ = 14.06%). The full model, incorporating interactions, accounts for 48.14% of the observed variability, suggesting the presence of an interaction between region and tissue (Fig. 3b).

## Evolutionary relationships of animal-associated viruses

### Flaviviridae

**Fig. 4:**
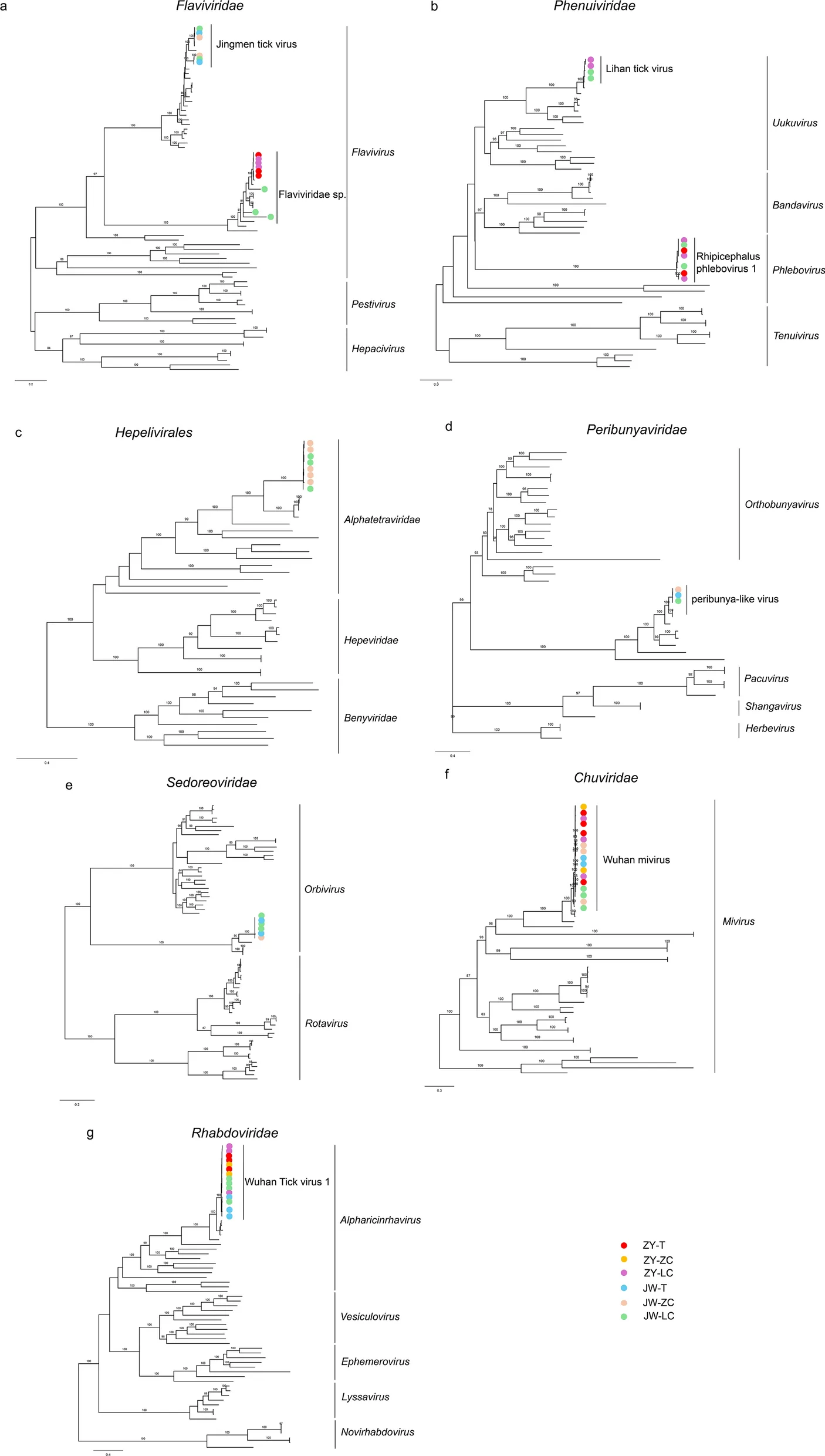
Phylogenetic trees of seven major families of animal-associated RNA viruses. Phylogenetic trees were inferred for each family of RNA viruses based on nucleotide sequences of the RNA-dependent RNA polymerase gene

A total of 83 tick-associated flavivirus sequences were identified in the tick pools examined. Phylogenetic analysis, based on representative virus sequences, revealed that a subset of these sequences exhibited a high degree of relatedness to the Rhipicephalus-associated flavivirus, with genomic sequence similarity ranging from 88.2 to 98.4% (Fig. 4a). A secondary group was linked to the JMTV. Following its initial discovery from *R. microplus* in Hubei Province [13], the tick has been identified in various regions globally and has been linked to a diverse array of hosts, including humans. Phylogenetic analysis based on nucleotide sequences of segment 1 demonstrated that the JMTV sequences identified in the present study were closely clustered with other JMTV sequences detected in Yunnan, Guangxi, Wenzhou, Japan, and Laos. These sequences have been classified as belonging to Phylogenetic Group Ⅳ, as previously reported in the literature [42].

### Phenuiviridae

Phylogenetic analysis indicates that the strains from this study are closely related to phleboviruses found in *R. microplus* ticks in Yunnan, with sequence similarities ranging from 95.1 to 99.3% [43] (Fig. 4b). While the number of species is rapidly increasing, many viruses are restricted to single regions, highlighting the unclear diversity and potential risks of tick phleboviruses. Moreover, our analysis revealed the presence of several Lihan tick virus sequences in samples originating from both imported and local sources. These viruses, which are predominantly prevalent in *R. microplus* ticks across various countries, have the capacity to infect birds, mammals, and humans [44, 45]. However, they are not currently regarded as a significant public health concern. This study lends further credence to their distribution in Yunnan ticks.

### Hepelivirales sp.

The presence of *Hepelivirales* sp. in the order *Hepelivirales* was identified exclusively in imported samples and exhibited a high positive rate (60%). Phylogenetic analysis based on the sequences of the putative polyprotein revealed that the virus clustered with *Hepelivirales* sp. strains found in *Haemaphysalis longicornis* in Shandong Province, China [46], and formed a separate branch in the phylogenetic tree (Fig. 4c). The polyprotein exhibited 74.78% nucleotide identity with the closest species, *Hepelivirales* sp. isolate CLCM-019 (GenBank accession no. OR114698), suggesting the potential for novel virus classification.

### Peribunyaviridae

A novel peribunya-like virus was identified in imported samples, and these viruses were found in all three different types of tissues. Phylogenetic analysis based on the putative RdRp gene revealed that JW-2 clustered with three other peribunya-like virus strains found in *R*. *microplus* in Hainan Province and other regions in China (Fig. 4d). The viruses in question exhibited nucleotide identity of 74.66% with the RdRp gene of the closest peribunya-like virus strains, which were found in other species of tick, including *Dermacentor everestianus* and *Hyalomma asiaticum*, in the northern west of China (PQ605445, ON746492). This finding suggests a potential new role for ticks as viral vectors.

### Sedoreoviridae

The presence of Sedoreoviridae-related sequences was exclusively detected in imported samples from all three types of tissues. A subsequent analysis, employing a phylogenetic tree constructed based on the nucleotide sequences of Segment 3 (VP3), revealed that the virus strains formed a distinct branch. The sequence of VP3 exhibited a 71.73% nucleotide similarity with the most closely related virus strain, St Croix River virus isolate HN3W/2022, which was identified in Hainan Province, China, from *R*. *microplus*. The isolate in question belongs to the genus *Orbivirus*, suggesting the potential for a novel virus. This finding is consistent with the species demarcation criteria of the genus *Orbivirus* (VP3 nucleotide identity of < 76%), as delineated by the International Committee on Taxonomy of Viruses (ICTV; Fig. 4e). Although the St. Croix River virus was isolated from ticks in 2001 and can be transmitted through the ovaries, its pathogenicity has not been studied [47].

### Chuviridae

The Wuhan mivirus was identified in both imported and local samples of three types of tissues belonging to the genus *Mivirus*, family Chuviridae. Phylogenetic analysis based on the putative RdRp gene revealed that virus sequences in our study cluster with other strains detected in the northwest and central areas of Yunnan from *R. microplus* [10] (Fig. 4f), indicating the wide distribution of this virus in Yunnan Province.

### Rhabdoviridae

With the exception of 11 unclassified Rhabdoviridae-related sequences, viral sequences belonging to the family Rhabdoviridae, genus *Alpharicinrhavirus*, were exclusively obtained from the tissue of salivary glands and ovaries. The construction of a phylogenetic tree based on the nucleotide sequences of the L gene indicated that these sequences grouped with other strains of Wuhan tick virus 1 (Fig. 4g). These strains were identified in various regions of China, exhibiting nucleotide identities ranging from 97.3 to 99.1%.

## Phylogenetic analyses of bacterial genera

For a further phylogenetic identification of the taxa of interest, maximum likelihood (ML) phylogenetic analysis of the genera *Coxiella*, *Rickettsia*, *Anaplasma,* and *Ehrlichia* were performed. The analysis was based on the obtained ASVs assigned to these genera, and the top-hit homologous sequences downloaded from GenBank.

**Fig. 5:**
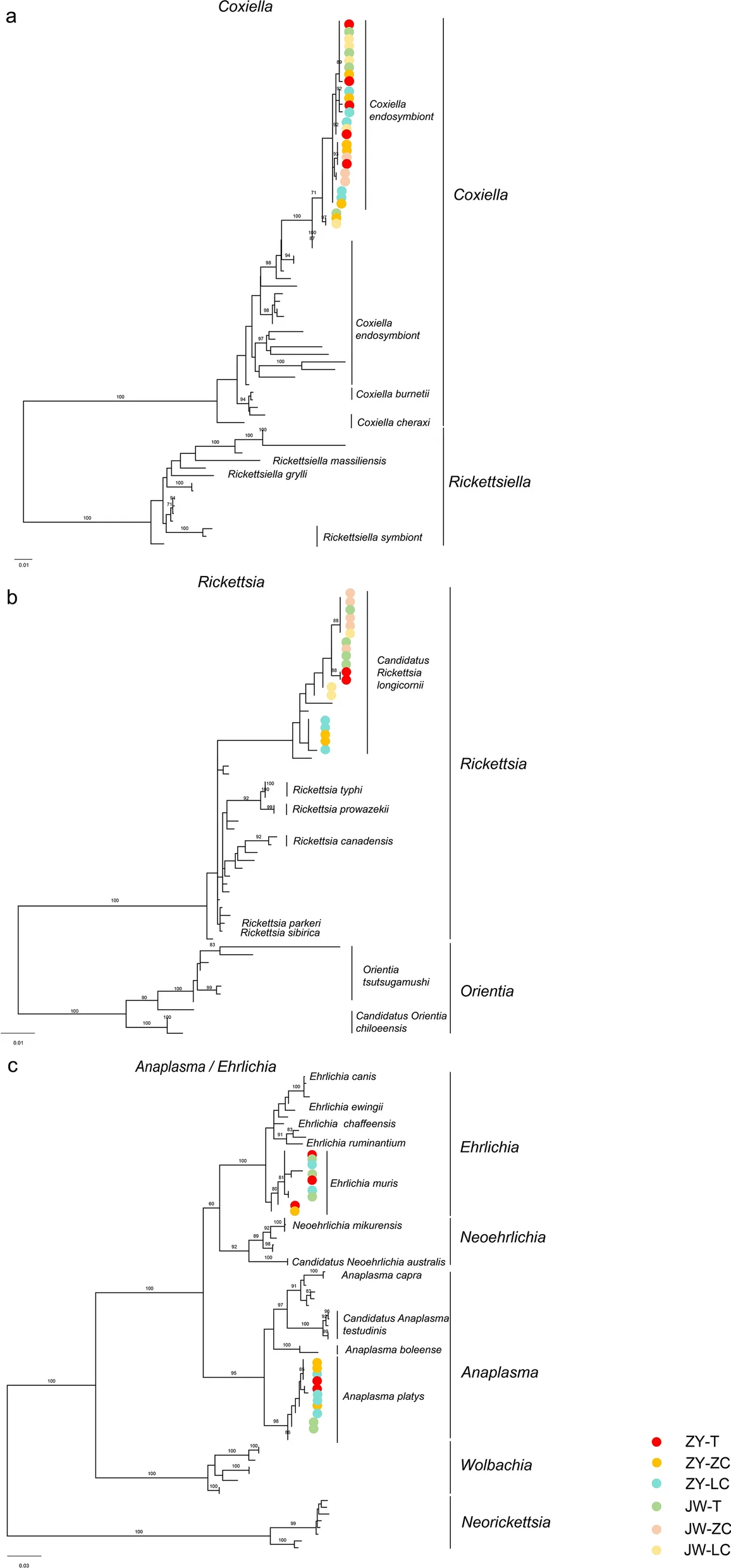
Phylogenetic trees of four bacterial genera were constructed using maximum likelihood inference and the GTR + I + G evolutionary model, based on a partial sequence of the 16S rRNA gene. The percentage of trees in which the associated taxa clustered together in the bootstrap test (1000 replicates) is shown next to the branches. **a** Genus *Coxiella*, 470 nt of the 16S rRNA gene. **b** Genus *Rickettsia*, 444 nt of the 16S rRNA gene. **c** Genera *Anaplasma* and *Ehrlichia*, 444 nt of the 16S rRNA gene

Coxiella-related ASVs were identified in both imported and local samples of three types of tissues belonging to the genus *Coxiella*, which include known pathogens such as *Coxiella burnetii*, the causative agent of Q fever in a variety of vertebrate species. The phylogenetic analysis showed that 28 representative ASVs identified in the genus *Coxiella* clustered with other Coxiella endosymbionts associated with *Rhipicephalus* ticks (Fig. 5a), exhibiting nucleotide identities ranging from 99.15 to 99.57% with closely related isolate Rhmicro1 (KP994839).

Rickettsia-related ASVs were identified in both imported and local samples of three types of tissues. A phylogenetic tree was constructed composed of 19 representative ASVs classified as *Rickettsia*, *Rickettsia* ASVs in our study clustered with each other and were closely related to the Candidatus *Rickettsia longicornii* isolate YBHC494-E7/2019 (MN997108), with high nucleotide sequence identities of 99.32% (Fig. 5b).

Phylogenetic analysis based on the representative ASVs classified as the genera *Anaplasma* and *Ehrlichia* belonging to the family Anaplasmataceae revealed that *Anaplasma*-related ASVs identified in our study clustered with other *Anaplasma platys* strains and were closely related to the isolate CTU/Vietnam/A.naDog-CT05 (PP809056) with high nucleotide sequence identities ranging from 99.32 to 99.77% (Fig. 5c). Additionally, *Ehrlichia*-related ASVs in our study clustered and matched (99.10 to 99.55% identity) to different species in the genus *Ehrlichia*, including *Ehrlichia ewingii*, *Ehrlichia muris*, *Ehrlichia pampeana*, and *Ehrlichia shimanensis* (Fig. 5c).

## Bacterial diversity association with tissue and location

### Alpha diversity

**Fig. 6:**
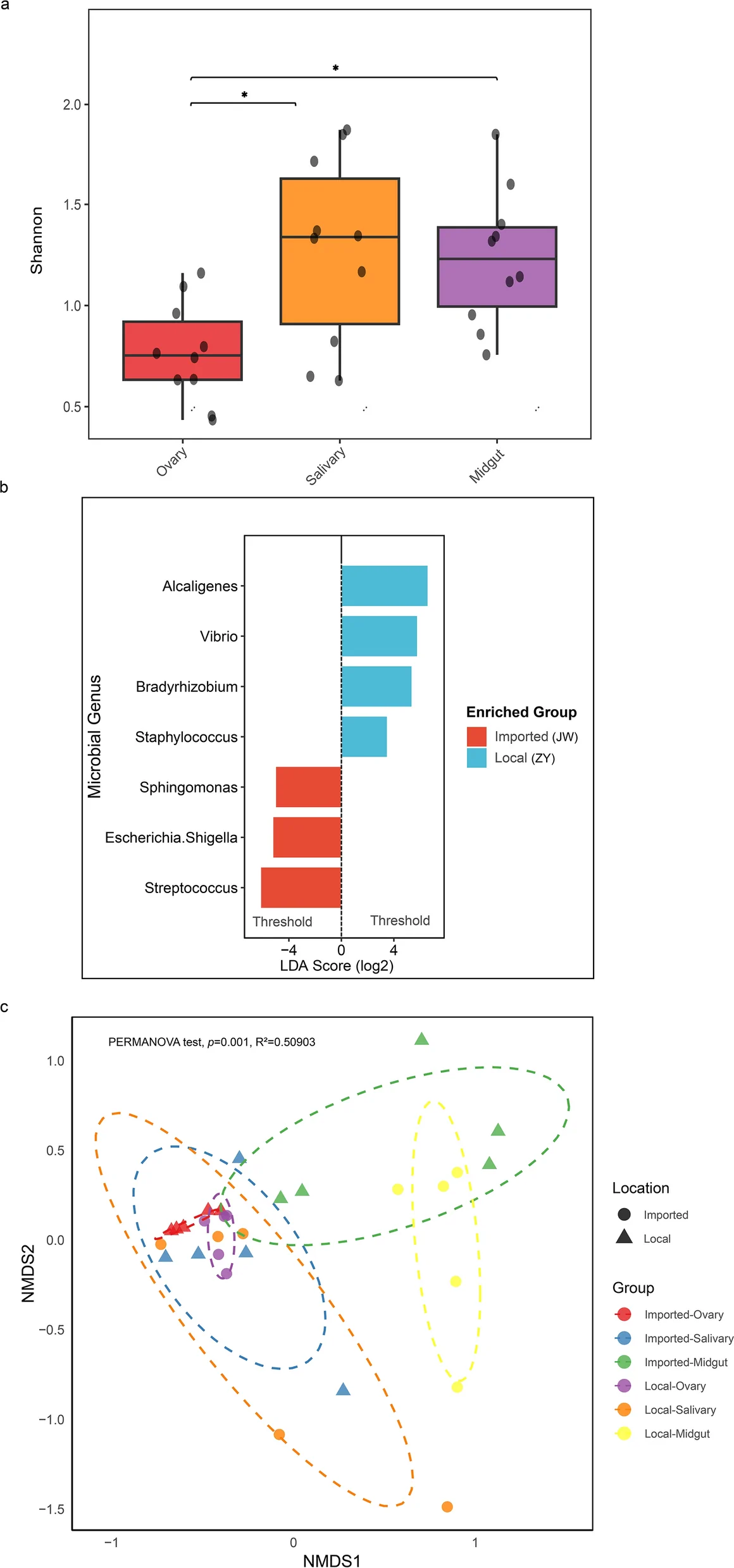
Regional and tissue variation of the microbiome. **a** Tissue differences in total bacterial diversity (Shannon diversity index). Kruskal–Wallis tests were performed for each pairwise comparison, *P < 0.05. **b** Bacterial clusters (biomarkers) contributing to regional differences were identified by linear discriminant analysis effect size analysis. LDA score (log) > 2. **c** Non-metric multidimensional scaling analysis displays the variations in bacterial composition between region and tissue. In the NMDS plots, the circles represent the 95% normal probability ellipse

There was no statistically significant difference in the Shannon index (Wilcoxon rank-sum test, *p* = 0.101) between the imported and local samples (Additional file 1: Fig. S2a). At the tissue level, a series of pairwise comparisons were conducted on the three tissues (ovary, salivary gland, and midgut) (Fig. 6a). These comparisons indicated that there were significant differences in Shannon's index between the ovary and the salivary gland/midgut (Kruskal–Wallis test, *H* = 9.494, df = 2, *p* = 0.00868, Wilcoxon rank-sum test for pairwise comparisons, *p* = 0.017, *p* = 0.012), while no significant differences were observed between the salivary gland and the midgut. The samples from the salivary gland demonstrated the highest Shannon diversity index, while the ovary exhibited the lowest. Region–tissue combination groups revealed significant differences among the six combination groups (Kruskal–Wallis test, *p* = 0.0119). Pairwise comparisons revealed significant differences between local-midgut and imported-ovary (Wilcoxon rank-sum test, *p* = 0.0397) and between imported-midgut and local-ovary (Wilcoxon rank-sum test, *p* = 0.0397), while no significant differences were observed among other combinations (Additional file 1: Fig. S2b).

### LEfSe analysis

The LDA effect size (LEfSe) analysis was conducted to further identify virus clusters (biomarkers) that were major contributors to the differences between the sample regions. LEfSe analysis (LDA score (log) > 2) revealed that the local group (ZY) was significantly enriched in the following genera: *Alcaligenes*, *Vibrio*, *Bradyrhizobium*, and *Staphylococcus*. The imported group (JW) was found to be significantly enriched in the following genera: *Sphingomonas*, *Escherichia*, and *Streptococcus* (Fig. 6b).

### Beta diversity

The impact of location on microbial community structure was not significant (PERMANOVA test, *p* = 0.101, *R*^2^ = 0.06595). Tissue exhibited a highly significant effect on microbial community structure (PERMANOVA test, *p* = 0.001, *R*^2^ = 0.42738), explaining a high proportion of the variation (42.7%). The interaction between location and tissue (Location–Tissue) was also found to be highly significant (PERMANOVA test, *p* = 0.001, *R*^2^ = 0.50903), indicating that microbial community structures vary between tissues in different regions (Fig. 6c).

### The correlation of diversity between virome and bacteria

**Fig. 7:**
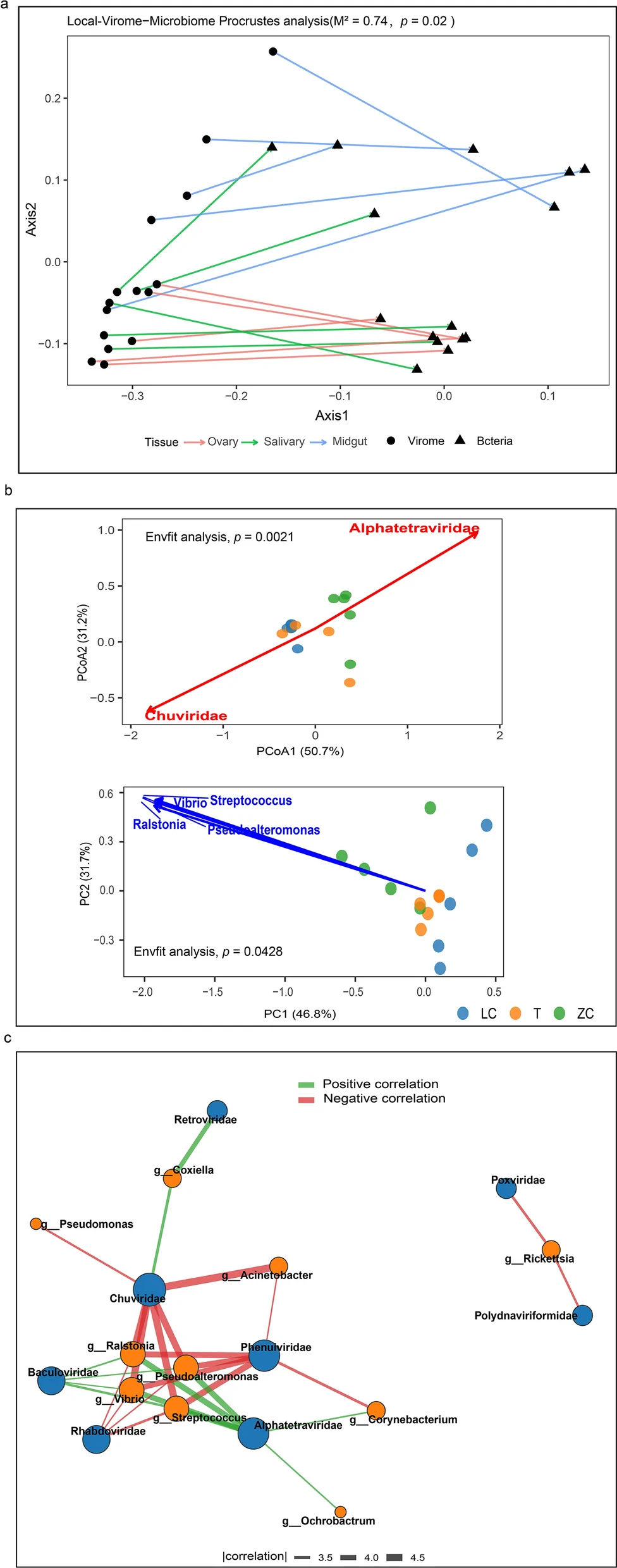
Virome–bacteria correlation analysis. **a** Virome–bacteria Procrustes analysis of local samples. **b** Key driving factors of Virome–bacteria correlation. **c** Virome–bacteria association network of local samples

A correlation analysis was subsequently performed on the samples of virome and bacteria. The Procrustes analysis revealed no substantial overall correlation in diversity between the virome and bacteria (*M*^2^ = 0.9526, *p* = 0.437, Additional file 1: Fig. S3). However, local sample groupings demonstrated an association between the viral and bacterial communities (Procrustes analysis, *M*^2^ = 0.7402, *p* = 0.02, Fig. 7a). Key drivers of viral–bacterial interactions were identified, with Alphatetraviridae and Chuviridae significantly impacting bacterial community structure (envfit analysis, *p* = 0.0021, *P* = 0.0428, Fig. 7b). In contrast, genera *Vibrio*, *Streptococcus*, *Ralstonia*, and *Pseudoalteromonas* exhibited a substantial impact on viral community structure (envfit analysis, *p* < 0.05). A subsequent virus–bacterial correlation analysis yielded 20 significant virus–bacteria pairs, meeting the criteria of |*r*|> 0.5 and *p* < 0.05. The key hub virus family was identified as Chuviridae (connectivity = 7), followed by Phenuiviridae and Alphatetraviridae (connectivity = 6). The predominant bacterial genera identified included *Vibrio*, *Streptococcus*, *Ralstonia*, and *Pseudoalteromonas* (connectivity = 5, Fig. 7c).

## Discussion

This study employed meta-transcriptomic sequencing to analyze viruses and bacteria carried by *R. microplus* from Pu'er and Xishuangbanna, in Yunnan Province. A total of 63 viral families were identified, 28 of which were linked to animals. Of particular interest is the prevalence of zoonotic viral families, such as Flaviviridae, Phenuiviridae, and Peribunyaviridae. The wide distribution of phleboviruses and JMTV is a matter of concern due to their potential to pose public health hazards in this region. In recent years, JMTV has been rapidly identified worldwide across diverse hosts, spanning 27 countries and infecting 7 vertebrate and 11 arthropod species [48]. This broad ecological range has driven significant viral diversity, though the underlying mechanisms warrant further investigation. Additionally, the number of species belonging to the family *Phenuiviridae* is rising rapidly, comprising 23 genera and 159 species, several studies identify new viruses belonging to the genus *phlebovirus*, with the majority of human-associated phleboviruses generally associated with sandflies and mosquitoes, such as Rift Valley fever virus [49] and Toscana virus [50]. However, several newly discovered tick-borne phleboviruses, with potential risk, prompt us to further strengthen monitoring.

Furthermore, our comprehensive investigation led to the identification of several previously unclassified viruses, underscoring the adaptability and potential transmission capacity of these viruses. The novel orbivirus-like virus (family Sedoreoviridae), detected exclusively in imported *R. microplus* ticks, suggests possible cross-border introduction via infested cattle or migratory ungulates. Phylogenetically, they cluster with St. Croix River virus (SCRV), a distantly related tick-associated species within the genus *Orbivirus* originally isolated from *Ixodes scapularis* eggs [47]. SCRV can establish persistent infections and undergo transovarial transmission in ticks, a trait that may also apply to this novel virus. The genus *Orbivirus* includes several important pathogens that caused large outbreaks in domestic animals, such as bluetongue virus (BTV), African horse sickness virus (AHSV), and epizootic hemorrhagic disease virus (EHDV), leading to high mortality in sheep, cattle, horses, and deer [51]. Although tick‑borne orbiviruses like Chenuda virus, Chobar Gorge virus, and Great Island virus have been gradually described [52], their pathogenicity remains unclear, and little is known about such viruses in Yunnan. Given the pathogenic potential of orbiviruses, further surveillance and experimental studies are warranted. In addition, the novel peribunya‑like virus further expands our understanding of peribunyavirus diversity and host range. Most peribunyaviruses are primarily associated with mammals, birds, mosquitoes, culicoids, and psychodid sandflies [53]; yet their detection in ticks is increasingly documented. For instance, Ngari virus in Guinea, Desert peribunya‑like virus in Xinjiang, and Hubei tick peribunyavirus in central China have been detected in various tick species (e.g., *Amblyomma*, *Rhipicephalus*, *Hyalomma*, and *Haemaphysalis*) [54–56], indicating that peribunyavirus transmission ecology may be more complex than previously recognized. Thus, more studies are urgently needed to assess the public and veterinary health risks posed by this novel tick‑associated peribunyavirus in Yunnan's border regions. Finally, the detection of novel Hepelivirales sp. and its close relatives in ticks from multiple provinces in China (Shandong, Hebei, and Yunnan) [46, 56] indicates its extensive distribution across diverse tick species and geographical regions in China. Given that the order *Hepelivirales* also contains vertebrate pathogens such as hepatitis E virus, the cross-species transmission and pathogenic potential of this novel virus warrant close attention.

In addition, a total of 167 bacterial genera were identified, including several potential pathogens, such as the genera *Coxiella*, *Rickettsia*, *Anaplasma*, and *Ehrlichia*. Our study revealed a high prevalence and relative abundance of Coxiella endosymbionts closely related to *Coxiella burnetii*, the causative agent of Q fever, across all tick samples in our study. This finding aligns with the documented widespread circulation of diverse *Coxiella* strains in ticks globally, many of which are maternally inherited endosymbionts [57]. The close phylogenetic proximity and high prevalence in ticks highlight a potential reservoir for pathogen evolution or emergence [58]. And the concurrent detection of *C. burnetii* in wild lagomorphs and their ticks in endemic areas underscores the zoonotic risk associated with *Coxiella* circulation in tick populations [59]. Further studies should assess the vector competence of local ticks and the potential for these endosymbionts to acquire virulence traits. Additionally, *Rickettsia* spp. are significant arthropod-borne pathogens possessing considerable zoonotic potential [60]. The high prevalence of *Rickettsia longicornis* sequences in our study also deserves attention, which is the predominant spotted fever group. *Rickettsia* in South Korea, demonstrating a 31.2% positivity rate in ticks from Korean water deer [61], and has also been detected in China and Japan [62]. Notably, its detection rate in western China is significantly higher than in eastern regions, suggesting distinct geographical distribution patterns [63]. Although its pathogenicity requires further clarification, the high prevalence and broad geographical range of *R. longicornis* warrant its prioritization in future surveillance.

The findings of this study are consistent with the results of recent tick microbial studies in China, which demonstrate a high degree of pathogen diversity associated with particular tick species and ecological settings [18, 46, 64]. A salient finding was the considerably elevated viral diversity observed in imported samples from Yunnan in comparison to local samples (Shannon index, *p* < 0.05). The phenomenon may be attributed to various factors, including cross-border animal movement, increased biodiversity among hosts, and the presence of unique microclimates, as observed in the Xinjiang and Ningxia provinces [18, 20, 55]. The influx of migratory hosts and international trade have been identified as factors that likely introduce diverse viral populations, thereby increasing the risk of zoonotic spillover. For instance, a cross-border transmission genotype of SFTSV was identified in ticks from Myanmar, and unique viruses such as Sedoreoviridae were detected in imported samples [15]. The analysis of β-diversity indicated that geographic location accounted for 14.06% of the variation in viral diversity (*p* < 0.05), while tissue type contributed 21.56%, with the midgut showing the highest viral diversity. These findings underscore the imperative for ongoing surveillance of tick-borne pathogens in Yunnan's border regions to address emergent public health risks.

Furthermore, the tissue specificity of bacterial communities was more pronounced (PERMANOVA test, *R*^2^ = 42.7%, *p* = 0.001), with bacterial diversity in the salivary glands and midgut being significantly higher than in the ovaries (*p* < 0.05). This phenomenon may be associated with immune suppression or vertical transmission mechanisms. This tissue-specific distribution has the potential to exert a substantial influence on the transmission capacity of pathogens. For instance, the enrichment of pathogens in the salivary glands of infected ticks may enhance the efficiency of transmission through biting, while infection in the ovaries of the tick may lead to vertical transmission, thereby allowing pathogens to persist in the tick population [65, 66]. While the bacterial community differences between imported and local samples were not significant, LEfSe analysis revealed imported samples to be enriched in *Streptococcus* and *Escherichia–Shigella*, while local samples were dominated by *Vibrio* and *Bradyrhizobium* (Fig. 6b). This discrepancy could be indicative of variations in host between the two locations.

The vector competence of ticks can be modulated by complex virus–bacteria interactions through multiple mechanisms, including the production of antiviral metabolites by certain symbiotic bacteria that directly inhibit viral replication; competition between pathogens for limited cellular resources and ecological niches within tick tissues; and immune modulation, wherein co-infection alters tick immune responses [67–70]. Procrustes analysis revealed that the overall correlation between viral and bacterial communities was not significant. However, a substantial structural correlation was observed in local samples, suggesting that geographical locations may influence virus–bacteria interactions. Network analysis revealed significant negative correlations between Chuviridae viruses and several bacterial genera, implying possible antagonistic interactions between viruses and bacteria. Such interactions may play an important role in regulating the microbial balance within ticks and the transmission of pathogens. While these findings are preliminary and require further mechanistic validation, they provide valuable clues and lay the groundwork for future studies aimed at elucidating the specific roles of virus–bacteria interactions in tick vector competence.

## Conclusions

In summary, this study characterized the viral and microbial communities of *R. microplus* in different geographical locations in border areas of Yunnan. Three potential novel viruses, including Hepelivirales sp., a peribunya-like virus, and a Sedoreoviridae-like virus were identified. Our results indicated viral diversity was higher in imported samples than in local samples, while tissue-specific analysis revealed the midgut had the highest viral diversity. Beta diversity indicated that both geographic origin and tissue type influenced viral and bacterial community structures. Although overall virus–bacteria community correlation was nonsignificant, significant associations were detected in local samples, with network analysis revealing interactions between key viral families (e.g., Chuviridae) and bacterial genera. These findings offer insights into microbial diversity and zoonotic risk and support public health risk assessment of tick-borne diseases in the region.

## Supplementary Information

Additional file 1.

Additional file 2.

Additional file 3.

## Data Availability

The datasets supporting the findings of this article are available in the NCBI Sequence Read Archive (SRA) database (BioProject number: PRJNA1336877, PRJNA1335530).
