# Supplementary material for: A cross-border comparison reveals expanded microbial diversity and zoonotic risk in Rhipicephalus microplus ticks of Yunnan, China
Source: Parasit Vectors. 2026 Aug 13;19:485. doi: 10.1186/s13071-026-07572-1 (PMC13749989; doi:10.1186/s13071-026-07572-1)
Supplement: Supplementary file 1 [file 13071_2026_7572_MOESM3_ESM.docx]

**Figure S1.** Regional and tissue variation of the virome. Kruskal-Wallis tests were performed for each pairwise comparison, * P < 0.05. (a) Tissue differences in total virus diversity (Shannon diversity index). (b) Regional-Tissue differences in total virus diversity (Shannon diversity index).

**Figure S2.** Regional and tissue variation of the microbiome. Kruskal-Wallis tests were performed for each pairwise comparison, * P < 0.05 (a) Geographical difference in total bacterial diversity (Shannon diversity index). (b) Regional-Tissue differences in total bacterial diversity (Shannon diversity index).

**Figure S3.** Virome-bacteria Procrustes analysis of the correlation between virus and bacteria diversity.

**Figure S4.** Phylogenetic tree of the genus *Coxiella* constructed based on 470 nt of the 16S rRNA gene.

**Figure S5.** Phylogenetic tree of the genus *Rickettsia* constructed based on 444 nt of the 16S rRNA gene

**Figure S6.** Phylogenetic tree of the genera *Anaplasma* and *Ehrlichia* constructed based on 444 nt of the 16S rRNA gene.
